# Novel Oxovanadium Complex VO(hntdtsc)(NPIP): Anticancer Activity and Mechanism of Action on HeLa Cells

**DOI:** 10.3389/fphar.2020.608218

**Published:** 2021-02-08

**Authors:** Yinliang Bai, Honghua Zhang, Yali Wang, Longqing Zhu, Tao Shi, Hangzhi Wei, Jiyuan Xiao, Youcheng Zhang, Zhen Wang

**Affiliations:** ^1^ Department of Pharmacy, Lanzhou University Second Hospital, Lanzhou, China; ^2^ School of Pharmacy, Lanzhou University, Lanzhou, China; ^3^ Department of General Surgery, Lanzhou University Second Hospital, Lanzhou, China

**Keywords:** oxovanadium (IV) complexes, VO(hntdtsc)(NPIP), antiproliferative activity, mitochondrial-dependent apoptosis, HeLa xenograft

## Abstract

Oxovanadium complexes, particularly vanadyl (IV) derivatives with hybrid ligands of Schiff base and polypyridyl, have been demonstrated to possess great anticancerous therapeutic efficacy. However, most of the studies on the activity of these oxovanadium complexes have mainly focused on *in vitro* studies, and animal studies *in vivo* are extremely scarce. Based on the antitumor test results of four novel oxovanadium complexes in our previous work, this work further conducted a comprehensive antitumor activity study *in vitro* and *in vivo* on VO(hntdtsc)(NPIP), which owned the strongest inhibitory activity *in vitro* on multiple tumor cell proliferation. The cellular mechanism study suggested that VO(hntdtsc)(NPIP) inhibited the cell proliferation *via* arresting the cell cycle at G0/G1 phase through the p16-cyclin D1-CDK4-p-Rb pathway and inducing cell apoptosis through mitochondrial-dependent apoptosis pathway on HeLa cells. Inconsistent with the effects *in vitro*, VO(hntdtsc)(NPIP) significantly inhibited the growth of tumor and induced the apoptosis of cancer cells in mice xenograft models according to the results of nude mice *in vivo* image detection, H&E pathological examination, and immunohistochemical detection of p16/Ki-67 protein expression. Collectively, all the results, particularly studies *in vivo,* demonstrated that VO(hntdtsc)(NPIP) hold a potential to be the lead compound and further to be an anticervical cancer drug.

## Introduction

Malignant tumor is a serious threat to human health and is a disease with the significant mortality rate. In the late 1960s, the discovery of the anticancer effect of cisplatin and its application clinically opened up a new field of developing metal complexes as efficient anticancer drugs and set off a boom in the metal antitumor drugs research (Wong and Giandomenico, 1999; Kelland, 2007). In recent years, metal antitumor drugs have been widely used as the main chemotherapeutics; however, severe drug resistance and side effects, such as nephrotoxicity and neurotoxicity, seriously restrict its application in clinical practice (Siddik, 2003; Galluzzi et al., 2012). Thus, investigating novel transition metal anticancer drugs with low toxicity, high efficiency, and good bioavailability is imperative. Notably, some metal complexes with various ligands show more possibilities for the development of high-efficiency and low-toxicity anticancer drugs due to their good water solubility and low toxicity towards normal organisms. For years, a majority of achievements have been obtained for metal complexes, including ruthenium (Süssfink, 2010), tin (Kaluđerović et al., 2010), titanium (Meléndez, 2002), germanium (Pi et al., 2013), copper (Quassinti et al., 2013), palladium (Ulukaya et al., 2011), and vanadium (D'Cruz and Uckun, 2002). Among them, vanadium, an ultratrace element with interesting pharmacological properties present in animals and higher plants, has been extensively studied from structures to its various physiological functions.

**SCHEME 1:**
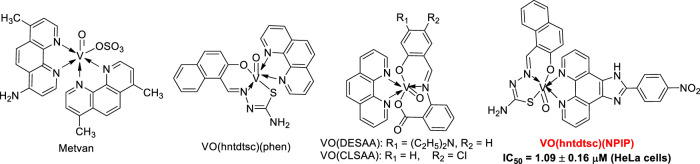
Structures of some classical oxovanadium complexes.

It is well known that vanadium has many different oxidation states, including +2, +3, +4, and +5 (Hirao, 1997; Bishayee et al., 2010). Particularly, tetravalent (IV) vanadyl (VO^2+^) derivatives, one of the most widely used analogues to investigate the pharmacological actions of vanadium, have been demonstrated to exert great therapeutic efficacy as anticancerous (Kioseoglou et al., 2015). Up to date, a series of oxovanadium complexes (IV) with different ligands have been designed and synthesized, and preliminary studies on their antitumor activities have been conducted as well (Scheme 1), suggesting that oxovanadium complexes have broad spectrum of antitumor potential, such as for cervical cancer, which is one of the leading causes of cancer death in women worldwide and poses a serious threat to the lives of patients and normal life (D'Cruz and Uckun, 2002; Zhang et al., 2013; Liao et al., 2014; León et al., 2015; Ying et al., 2015; Zeng et al., 2015). Schiff bases, one kind of typical ligands with chiral carbon atoms and abundant coordination patterns, could combine with transition metals to show a variety of biological activities, especially antitumor effects (Hazari et al., 2012; Ying et al., 2015). Particularly, thiosemicarbazones, as common derivatives of Schiff bases, were often chosen as ligands of vanadium due to their wide ranges of pharmacological applications (Benítez et al., 2011; Ying et al., 2015). However, current studies on thiosemicarbazones containing oxovanadium complexes mostly focus on the structure modification and the activity evaluation *in vitro*; it is extremely scarce in molecular mechanism studies and in animal models *in vivo*, which is a key stage in the transformation from experimental studies to clinical application (D'Cruz and Uckun, 2002; Hazari et al., 2012; Ying et al., 2015; Bai et al., 2018).

Growing evidence suggests that the interaction between transition metal complexes and nucleic acid molecules has a close correlation with their antitumor activity, so it is significant to investigate the interaction model of metal complexes with DNA for studying antitumor mechanism of metal complexes from molecular level (Wolkenberg and Boger, 2002; Sava et al., 2012). In our previous work, we have reported four novel thiosemicarbazones containing oxovanadium complexes, which had certain anticancer effects against HeLa, BIU-87, and SPC-A-1 cell lines (Bai et al., 2018). Among them, VO(hntdtsc)(NPIP) has been demonstrated to possess the strongest anticancer efficacy, especially for human cervical carcinoma HeLa cells via arresting the cell cycle and inducing cell apoptosis. Therefore, in order to further explore and identify the antitumor mechanism completely, the studies *in vitro* and *in vivo* on VO(hntdtsc)(NPIP) were carried out systematically in this study, and the results revealed that VO(hntdtsc)(NPIP) exerted antiproliferative activities which might be through arresting the cell cycle at G0/G1 phase via the p16-cyclin D1-CDK4-p-Rb pathway and inducing cell apoptosis via mitochondrial-dependent apoptosis pathway on HeLa cells.

## Materials and Methods

### Cell Culture

Human cervical cancer HeLa cell, human bladder cancer BIU-87 cell, human lung cancer SPC-A-1 cell, human stomach cancer SGC-7901 cell, human colon cancer HT-29 cell, human pancreatic cancer PANC-1 cell, and human hepatoma HepG2 cell were purchased from the Cell Bank of Type Culture Collection of the Chinese Academy of Sciences (Shanghai, China). All the above cancer cells were cultured in RPMI-1640 medium supplemented with 10% FBS, 100 U/ml penicillin, and 100 U/ml streptomycin at 37 °C with 5% CO_2_.

### Cytotoxicity Assay

In our previous work, we have synthesized four oxovanadium complexes and further characterized them by elemental analysis, UV-Vis, MS, IR, and NMR (Bai et al., 2018). In addition, the capacities of the four oxovanadium complexes and the corresponding free ligands to interfere with the growth of HeLa, BIU-87, and SPC-A-1 also have been evaluated by MTT assay. In this article, we further detected their ability to inhibit the growth of SGC-7901, HT-29, PANC-1, and HepG2. All the cell lines were incubated in RPMI1640 culture medium containing 10% fetal calf serum at 37 °C with 5% CO_2_. Exponentially growing tumor cells were seeded into a 96-well plate at a density of 1 × 10^5^ cells/ml after digestion with 0.25% trypsin. Compounds were then added to each cell with the final concentrations ranging from 0.1 to 200 μM. After incubation at 37 °C for 48 h, the medium was removed and 20 µl of MTT (0.5 mg/ml) were added to each well. The plates were then incubated at 37 °C for an additional 4 h to allow MTT to form formazan crystals, and subsequently 150 µl of DMSO was added into each well. The cell viability was determined by measuring the absorbance of each well at 490 nm using a Multiskan SSCENT microplate reader. IC_50_ values were determined by plotting the percentage viability vs. concentration on a logarithmic graph and reading off the concentration at which 50% of cells remain viable relative to the control.

### Evaluation of the ROS Level Changes by DCFH-DA Assay

HeLa cells were inoculated into 6-well plates at a density of 1 ×10^5^ cells per well with 2 ml culture medium and incubated in 37 °C with 5% CO_2_ for 24 h. Subsequently, VO(hntdtsc)NPIP at final concentrations of 0, 0.5, 1.0, and 2.0 μM were added to each well. After incubation for 48 h, the culture media were removed, and carboxy-21,71-dichloro-dihydro-fluoresceindiacetate probes (DCFH-DA) (Sigma, USA) dissolved in serum-free medium (1.0 ml) were added into each well at a final concentration of 10 μM and incubated for 20 min at 37 °C. Afterwards, HeLa cells were washed with cold PBS three times and analyzed with flow cytometer (Beckman, USA) at 488 nm for excitation and 525 nm for emission. The fluorescence intensity was quantified using NIS image processing system.

### Detection of the MMP by JC-1 Assay

HeLa cells were inoculated into 6-well plates at a density of 1 ×10^5^ cells per well and incubated in 37 °C with 5% CO_2_ for 24 h. Subsequently, VO(hntdtsc)NPIP at final concentrations of 0, 0.5, 1.0, and 2.0 μM were added to each well. After incubation for 48 h, the culture medium was removed and then 500 μl JC-1 incubation buffer was added at 37 °C for 20 min. Then the cells were observed under the fluorescence microscope with red fluorescence at 585 nm for excitation and 590 nm for emission, and green fluorescence at 514 nm for excitation and 529 nm for emission, respectively.

### Western Blot Assay

Protein preparation and western blotting analysis were performed using standard methods. HeLa cells were inoculated into 6-well plates at a density of 1 ×10^5^ cells per well and incubated at 37 °C with 5% CO_2_ for 24 h. VO(hntdtsc)NPIP at final concentrations of 0, 0.5, 1.0, and 2.0 μM were added to each well. After incubation for 24 h, the cells were digested with 0.25% trypsin and centrifuged for 5 min at 2000 rpm, then rinsed twice with ice-cold PBS, and lysed in 100.0 μl RIPA lysis buffer (Solarbio, R0010, China) containing 10% protease inhibitor for 30 min at 4 °C. The protein concentrations were determined by BCA protein assay kit (Solarbio, PC0020, China). Then, protein extracts (30 μg) were resolved in 8%–10% SDS-PAGE (SDS-PAGE; Bio-Rad, Hercules, CA, United States) and transferred to a polyvinylidene difluoride (PVDF) membrane (Macherey-Nagel). Subsequently, the PVDF membranes were blocked in 5% nofat milk for 1 h and incubated with primary antibodies Bax, Bcl-2, cytochrome c, cleaved caspase-3, cleaved caspase-8, cleaved caspase-9, p16, cyclin D1, CDK4, p-Rb, and β-actin with gentle rotation overnight at 4 °C and were next incubated with the secondary antibody consisting of horseradish peroxidase (HRP) conjugated for 2 h. The enhanced chemiluminescent (NEN Life Science Products, Boston, MA, USA) detection system was used for immunoblot protein detection.

### Animal Experiments

The 60 Kunming mice (half male and female) with a body weight of 22.0–24.0 g were provided by Medical Animal Experiment Center of Lanzhou University (SYXK (Gansu) 2013-0002), raised in a clean grade animal house. The 22 BALB/C nude mice (SPF level, female) with a body weight of 19.0–21.0 g were provided by Shanghai SLAC Laboratory Animal Co.Ltd (China, animal certificate no.: SCXK (Shanghai) 2012-0002). All procedures for the animal experiments were approved by the Experimental Animal Ethics Committee of Lanzhou University Second Hospital (no. d2019-004). The nude mice were raised in the SPF IVC animal room, and the feeds, bedding materials, cages, and contact instruments used were all used after high pressure disinfection. The animal room was maintained at a constant temperature (22 ± 2 °C), constant humidity (50 ± 10%), and noise was lower than 60 dB and was controlled by 12 h circadian rhythm. All animals were given adaptive feeding for 1 week after purchase and then were tested. We have tried our best to minimize the number and pain of animals.

### Acute Toxicity

Acute toxicity studies were carried out according to OECD guidelines (Oecd, 2008). Briefly, sixty 8-week-old Kunming mice weighing from 22 g to 24 g were purchased from the GLP Laboratory of Lanzhou University. The mice were divided into five groups randomly (*n* = 12, six female and six male mice). The mice in different groups were given intraperitoneal injection with VO(hntdtsc)(NPIP) aqueous solution at the doses of 4.0, 8.0, 16.0, 32.0, and 64.0 mg/kg, respectively. And the control group was given intraperitoneal injection with the same volume of water for injection. All treatments were operated by gavage with a single dose. The treated animals were observed continuously for the first 4 h followed by periodic monitoring for another 20 h. Then animals were observed once daily for a period of 14 days and the time of occurrence and recovery of toxic reactions (including animal hair, behavior, mental state, and excretion) was recorded. After 14 days, all animals were sacrificed to conduct histological studies on heart, lung, kidney, and liver by using H*&*E staining method.

### Establishment of HeLa Xenograft Model in Nude Mice

All studies involving animals are reported in accordance with the ARRIVE guidelines for reporting experiments involving animals. All procedures for the animal experiments were approved by the Experimental Animal Ethics Committee of the Second Hospital of Lanzhou University (no. d2019-004). Two nude mice (BALB/c-nu/nu, 8-week-old) were injected under the skin of the right lower neck axilla with 5 × 10^6^ HeLa cells in 0.2 ml PBS. When the tumors had reached an average volume of 500 mm^3^, the mice were anaesthetized by intraperitoneal injection of 10% chloral hydrate and then sacrificed by cervical dislocation. The subcutaneous tumor tissues were isolated by surgery in a room separated from the other animals and then were cut into equal-volume tumor pieces with a diameter of about 2.0 mm after removing the peripheral blood membrane and necrotic tissue. The 20 small pieces with the same or similar color, volume, and texture were selected and inoculated into 20 nude mice under the skin of the right lower neck axilla, respectively. The body weight of the nude mice was recorded daily after inoculation, and the long diameter and short diameter of the implanted tumor were measured. When the tumor volume reached 100.0 mm^3^, the mice were divided into four groups with five mice per group according to the random number table method, including the control group and high (4.0 mg/kg), medium (2.0 mg/kg), and low (1.0 mg/kg) dose groups containing VO(hntdtsc)(NPIP) at different concentrations, which was intraperitoneally injected, and the control group was intraperitoneally injected with water containing DMSO in the same volume. VO(hntdtsc)(NPIP) was administered every 3 days for a total of 7 times. During this period, the physiological conditions and weight changes of animals were observed every day, and the volume changes of transplanted tumors were measured with vernier calipers. Eventually, the body weight and tumor volume of the nude mice were statistically analyzed at the 0, 7th, 14th, 21th, and 28th day after tumor transplantation. The tumor volume (V) was calculated as V (mm^3^) = length (mm) × width (mm)2⁄2.

### Imaging Analysis of Xenografts *In Vivo*

The HeLa cell xenograft models of nude mice were successfully established according to the method in Section 2.8. On the 7th day after inoculation, nude mice were intraperitoneally injected with 200.0 μl luciferase substrate fluid D-luciferin potassium salt. After 10 min, the nude mice were deeply anesthetized in an isoflurane anesthesia box and transferred to an *in vivo* imager (Spectrum CT). The posture was fixed to fully expose the tumor. Bioluminescence signals were collected in the *in vivo* imaging system, and the corresponding light intensity was measured and analyzed. Repeat the procedure after the last administration on the 28th day after inoculation.

### Collection of Tumor Specimens and Preparation of Paraffin Sections

After the last *in vivo* imaging analysis, 10% chloral hydrate anaesthesia and cervical dislocation were performed in the nude mice, and the tumor xenografts were completely removed and placed in 4% paraformaldehyde fixative solution for 2 h. Then the tumor xenografts were dehydrated by graded ethanol solution at 4 °C. The gradient of ethanol is as follows: 70% (1 h), 80% (1 h), 90% (1 h), 95% (30 min), and 100% (30 min). Then the tumor xenografts were transparent as follows: 1/2 ethanol and 1/2 xylene (30 min), xylene (15 min + 10 min), and they were infiltrated as follows: 1/2 xylene and 1/2 paraffin (1 h, 55 °C), paraffin (1 h, 55 °C). The tissue was placed into a paraffin-filled mold for infiltration and then embedded and cooled in a paraffin embedding machine. Tissue wax blocks were marked and trimmed, and fixed on the paraffin-sectioning machine for continuous sectioning. The thickness of the slices was 3.0 μm. The slices were spread out over warm water and transferred into the glass slides. The slices were baked at 60 °C for 1 h and stored at room temperature after numbering for later use.

### Histopathological Evaluation by H&E Staining Assay

The prepared tissue sections were deparaffinized in xylene and rehydrated through graded ethanol solutions. The sections were stained in Harris hematoxylin solution for 5 min and then rinsed under running water and differentiated with 1% hydrochloric acid ethanol for 10 s. Following that, tissue sections were dehydrated in graded ethanol and permeabilized in xylene. Tissue sections were examined under an inverted optical microscope and the images were collected.

### TUNEL (FITC)/DAPI Double Staining Assay

The TUNEL staining assay was conducted using the *in situ* Cell Death Detection Kit from Roche Applied Science (Indianapolis, IN, United States), according to the manufacturer’s instructions. Briefly, paraffin-embedded tissue sections were deparaffinized using a standard protocol and rinsed with PBS; then, Proteinase K (20 mg/ml) solution was added after removing the surrounding liquid. After incubation for 20 min at room temperature, tissue sections were incubated with the TUNEL reaction buffer for 1 h at 37 °C in a humidified chamber under dark. Then tissue sections were stained with 4’,6-Diamidino-2-phenylindole dihydrochloride (DAPI) in the dark and observed by fluorescence microscopy at 461 nm and 520 nm, respectively.

### Immunohistochemical Staining

Tumor tissues were fixed in 10% neutral formalin and embedded in paraffin. Tissue sections were deparaffinized in xylene and rehydrated through graded ethanol solutions. Antigen retrieval was carried out using citric acid buffer solution. The sections were then transferred into 3% hydrogen peroxide solution and incubated for 10 min to eliminate endogenous peroxidase. Subsequently, the slices were rinsed in 0.01 M PBS and transferred to antibody sealing solution for incubation with 1 h. Tissue sections were stained using routine immunohistochemical techniques and primarily antibodies against Rabbit Anti-RatKi-67 and Rabbit Anti-Rat p16 monoclonal antibodies (the dilution ratios were all 1:1000) and secondary HRP-conjugated goat anti-rabbit IgG (H + L) (1:3000). The signal was detected using 3,3’N-Diaminobenzidine Tetrahydrochloride (DAB). Sections were counterstained with Mayer’s hematoxylin.

### Statistical Analysis

All experiments were carried out at least three times. Each sample was tested in triplicate. The results are expressed as percentage ± S.D. of control. Significance between the groups was determined by one-way analysis of variance (ANOVA), followed by Fisher’s PLSD procedure for post hoc comparison, to verify the significance between two means. *p* < 0.05 was considered significant for the all tests. The significance values were analyzed by SPSS 17.0 software.

## Results

### Antitumor Effect of VO(hntdtsc)NPIP *In Vitro*

#### Cytotoxicity Assay

**FIGURE 1:**
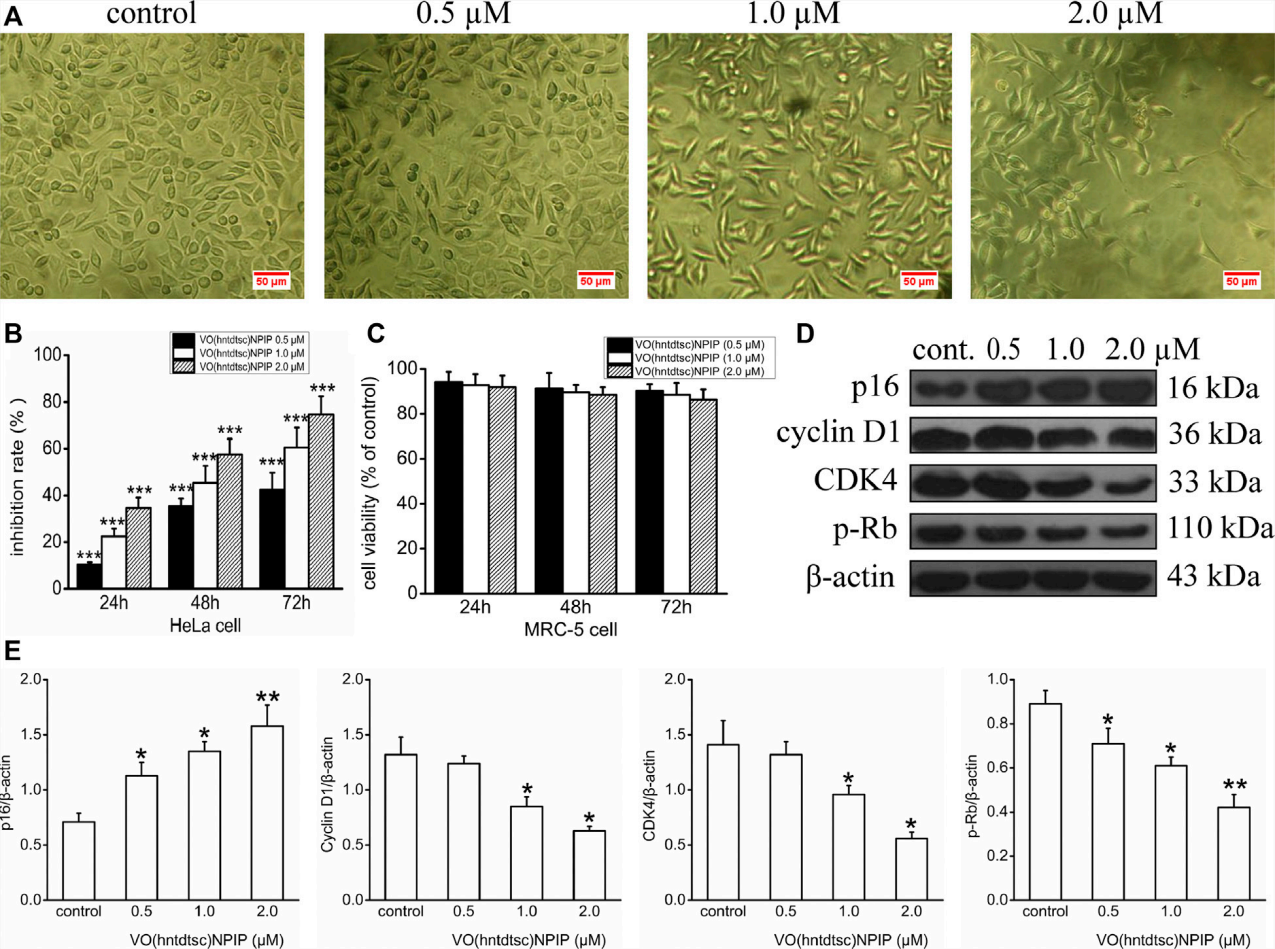
**(A)** The changes in the distribution of HeLa cells after being incubated for 48 h with VO(hntdtsc)NPIP at different concentrations (0, 0.5, 1.0, and 2.0 µM) (× 200). **(B)** The inhibition rates of VO(hntdtsc)NPIP on HeLa cells with different concentrations (0, 0.5, 1.0, and 2.0 µM) and time (24, 48, and 72 h). **(C)** The cell viability of MRC-5 cells after VO(hntdtsc)NPIP treatment with different concentrations (0.5, 1.0, and 2.0 µM) and time (24, 48, and 72 h). **(D)** Cell cycle regulatory proteins were detected using western blotting; HeLa cells were treated with VO(hntdtsc)NPIP at different concentrations (0, 0.5, 1.0, and 2.0 μM) for 24 h. **(E)** The density ratio of p16, cyclin D1, CDK4, and p-Rb to β-actin, after treatment with VO(hntdtsc)NPIP. Data are represented as mean ± SD of three independent experiments. *p < 0.05, **p < 0.01 or ***p < 0.001 compared to the control group.

In our previous work, preliminary cytotoxicity evaluation of four oxovanadium complexes had been conducted and the results showed that all the four oxovanadium complexes exhibited obvious cytotoxicity against HeLa, BIU-87, and SPC-A-1 cell lines. To further expand the antitumor spectrum of these oxovanadium complexes, cytotoxicity assays on SGC-7901, HT-29, PANC-1, and HepG2 cell lines were tested in this study and the results (Table 1) showed that the four oxovanadium complexes possessed certain cytotoxicity. Combining all cytotoxicity assays together, the oxovanadium complex VO(hntdtsc)(NPIP) had the best antiproliferative activity against the above-mentioned seven common human cancer cell lines, particularly HeLa cells. In order to further ascertain the antiproliferative efficacy, the proliferation inhibition rates of VO(hntdtsc)NPIP on HeLa cells at different concentrations (0, 0.5, 1.0, and 2.0 μM) were measured after 24, 48, and 72 h (Figure 1B). In detail, the inhibition rates of VO(hntdtsc)NPIP at 24, 48, and 72 h were as follows: 24 h: (10.37 ± 1.01) %, (22.55 ± 3.23) %, and (34.52 ± 4.61) %; 48 h: (35.33 ± 3.36) %, (45.30 ± 7.23) %, and (57.46 ± 6.69) %; and 72 h: (42.36 ± 7.33) %, (60.44 ± 8.63) %, and (74.61 ± 7.81) %. The results revealed that the significant antiproliferative efficacy of VO(hntdtsc)NPIP against HeLa cells was in a good concentration-dependent and time-dependent manner. In addition, after the incubation of HeLa cells by VO(hntdtsc)NPIP for 48 h, the cells observed in microscope field were less and sparsely distributed with increasing the concentration of the coordination compound (Figure 1A). In addition, the cytotoxicity assay of VO(hntdtsc)NPIP on the nontumor cell lines, MRC-5 cell lines, was also examined at different concentrations (0, 0.5, 1.0, and 2.0 μM) after 24, 48, and 72 h (Figure 1C), and the result revealed that VO(hntdtsc)NPIP exhibited no obvious cytotoxicity against MRC-5 cell lines. Therefore, VO(hntdtsc)NPIP was chosen for further investigation respecting its mechanism of anticancer activity on HeLa cells.

**Table 1:** Antiproliferative effects of four oxovanadium (IV) complexes and the corresponding free ligands and the positive drug cis-platin on different tumor cells lines after 48 h of treatment. Data are expressed as IC50 (μM).

Comp.	IC_50_ (μM)						
	HeLa	BIU-87	SPC-A-1	SGC-7901	HT-29	PANC-1	HepG2
Cisplatin	5.54 ± 0.81	7.82 ± 0.64	8.65 ± 1.01	7.32 ± 0.65	10.14 ± 1.02	13.32 ± 1.52	12.64 ± 1.22
Hntdtsc	100.04 ± 7.56	98.16 ± 6.78	74.56 ± 5.63	174.12 ± 16.35	110.23 ± 22.31	97.63 ± 6.89	169.36 ± 9.87
VO(acac)_2_	87.21 ± 7.24	76.87 ± 6.12	84.14 ± 5.89	123.69 ± 14.67	113.36 ± 23.14	134.69 ± 10.28	227.35 ± 36.58
CPIP	39.69 ± 7.12	60.23 ± 5.12	41.02 ± 3.45	101.23 ± 6.28	79.16 ± 13.46	98.41 ± 8.66	146.68 ± 14.38
NPIP	24.35 ± 1.63	39.65 ± 5.56	31.01 ± 5.01	79.75 ± 6.66	97.12 ± 10.25	90.46 ± 8.65	202.12 ± 26.46
MEPIP	41.02 ± 4.63	56.45 ± 7.36	61.02 ± 6.32	39.77 ± 5.22	110.03 ± 9.33	77.63 ± 6.11	59.38 ± 4.41
HPIP	78.62 ± 7.02	47.89 ± 4.12	54.36 ± 2.13	66.02 ± 7.47	198.22 ± 15.55	77.36 ± 8.12	99.69 ± 7.36
VO(L)MEPIP	20.11 ± 2.68	34.52 ± 3.67	24.63 ± 4.37	33.55 ± 1.99	51.22 ± 7.17	67.23 ± 4.34	56.56 ± 4.66
VO(L)NPIP	1.09 ± 0.16	4.51 ± 0.68	7.61 ± 0.55	14.23 ± 1.36	26.34 ± 4.11	35.23 ± 6.25	19.32 ± 3.45
VO(L)CPIP	10.36 ± 1.23	8.69 ± 1.05	21.43 ± 3.24	15.66 ± 2.33	31.23 ± 4.02	40.36 ± 2.99	33.31 ± 4.37
VO(L)HPIP	40.26 ± 4.01	19.86 ± 2.47	26.32 ± 4.75	54.10 ± 4.17	101.02 ± 8.15	69.22 ± 3.63	60.29 ± 4.44

* L = hntdtsc. Cells were treated with various concentrations (0.1, 0.5, 1.0, 5.0, 10.0, 50.0, 100.0, and 200.0 µM) of tested compounds for 48 h. Cell viability was determined by MTT assay and IC_50_ values were calculated as the mean ± SD of triplicate in three independent experiments.

#### Influence of VO(hntdtsc)NPIP on Cell Cycle

In order to define the mechanism of antiproliferative effect of VO(hntdtsc)NPIP on HeLa cells, the cell cycle-phase distribution was analyzed by flow cytometry with PI staining as described in our previous work (Bai et al., 2018). To further confirm the mechanism of VO(hntdtsc)NPIP on arresting cell cycle at G0/G1 phase, the expression of cell cycle regulatory proteins was detected using western blot assay. As shown in Figures 1D,E, VO(hntdtsc)NPIP treatment on HeLa cells could significantly increase the expression of p16 protein (*F =* 68.55, *p* < 0.001) and decrease the expression of cyclin D1, CDK4, and p-Rb (*F =* 19.15, *p* < 0.05; *F =* 20.41, *p* < 0.05; *F =* 75.46, *p* < 0.001) with concentrations increasing (0, 0.5, 1.0, and 2.0 μM).

### Evaluation of ROS Levels by DCFH-DA Assay

**FIGURE 2:**
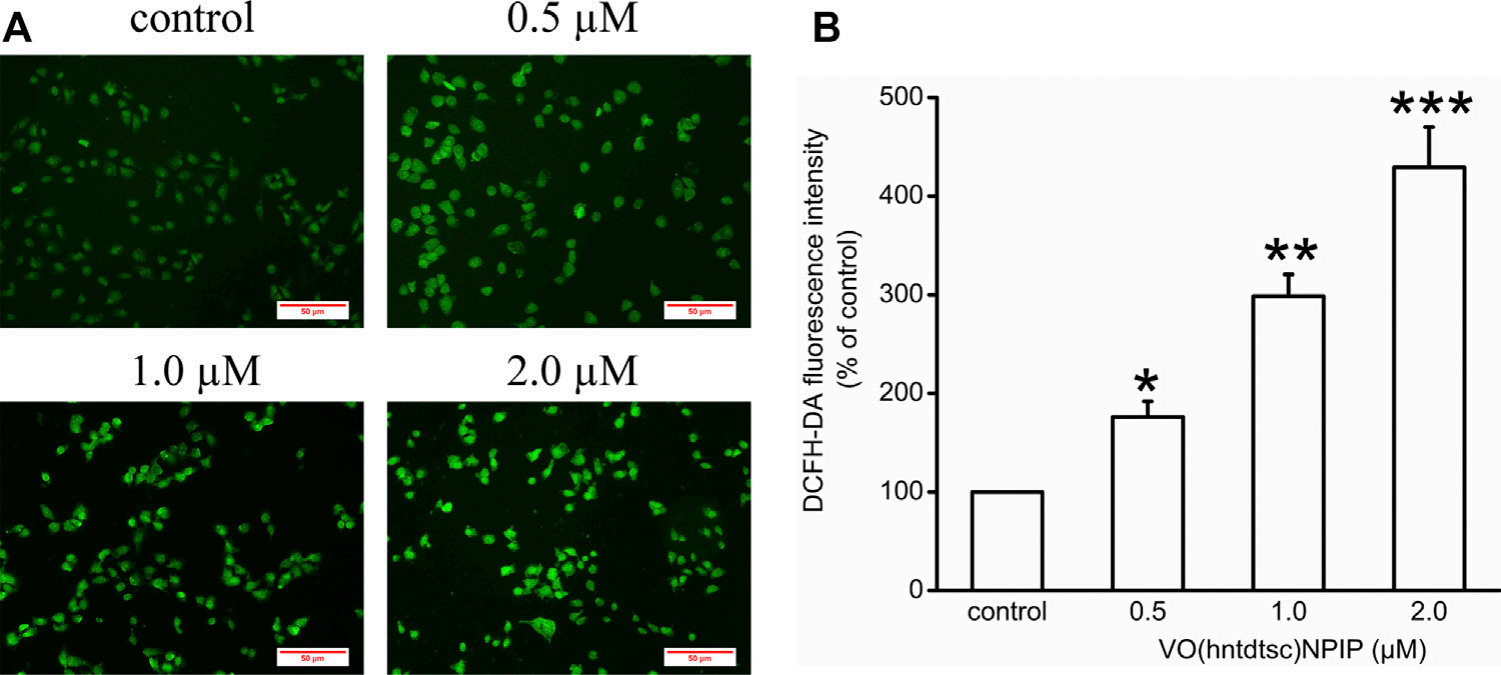
Effects of VO(hntdtsc)NPIP on the level of ROS in HeLa cells. **(A)** The production of ROS in HeLa cells after various concentrations (0, 0.5, 1.0, and 2.0 µM) of VO(hntdtsc)NPIP treatment for 48 h was detected by a fluorescence probe DCFH-DA (× 400). **(B)** Histograms displayed the fluorescence intensity after different concentrations of VO(hntdtsc)NPIP treatment. Data are represented as mean ± SD of three independent experiments. *p < 0.05, **p < 0.01 or ***p < 0.001 compared to the control group.

Our previous work has revealed that VO(hntdtsc)NPIP could effectively induce apoptosis of HeLa cells through increasing the apoptosis rates and changing the cell morphology, such as chromatin condensation and nuclear fragmentation (Bai et al., 2018). To further verify the mechanism of HeLa cells apoptosis induced by VO(hntdtsc)NPIP, we tested the efficacy of VO(hntdtsc)NPIP on ROS levels of HeLa cells at different concentrations (0, 0.5, 1.0, and 2.0 μM) by fluorometric assay using 2’,7’-dichlorofluorescein diacetate (DCFH-DA) as the fluorescence probe (Kobayashi et al., 2002). As shown in Figure 2, the green fluorescence was weak in the control group, while a stronger green fluorescence was observed in the VO(hntdtsc)NPIP treatment groups and the green fluorescence intensity in HeLa cells was enhancing with concentrations of VO(hntdtsc)NPIP increasing (0, 0.5, 1.0, and 2.0 µM), suggesting that VO(hntdtsc)NPIP could significantly induce the increase of ROS levels in HeLa cells in a dose-dependent manner (*F =* 112.24, *p* < 0.001).

#### Evaluation of Mitochondrial Membrane Depolarization by JC-1 Analysis

**FIGURE 3:**
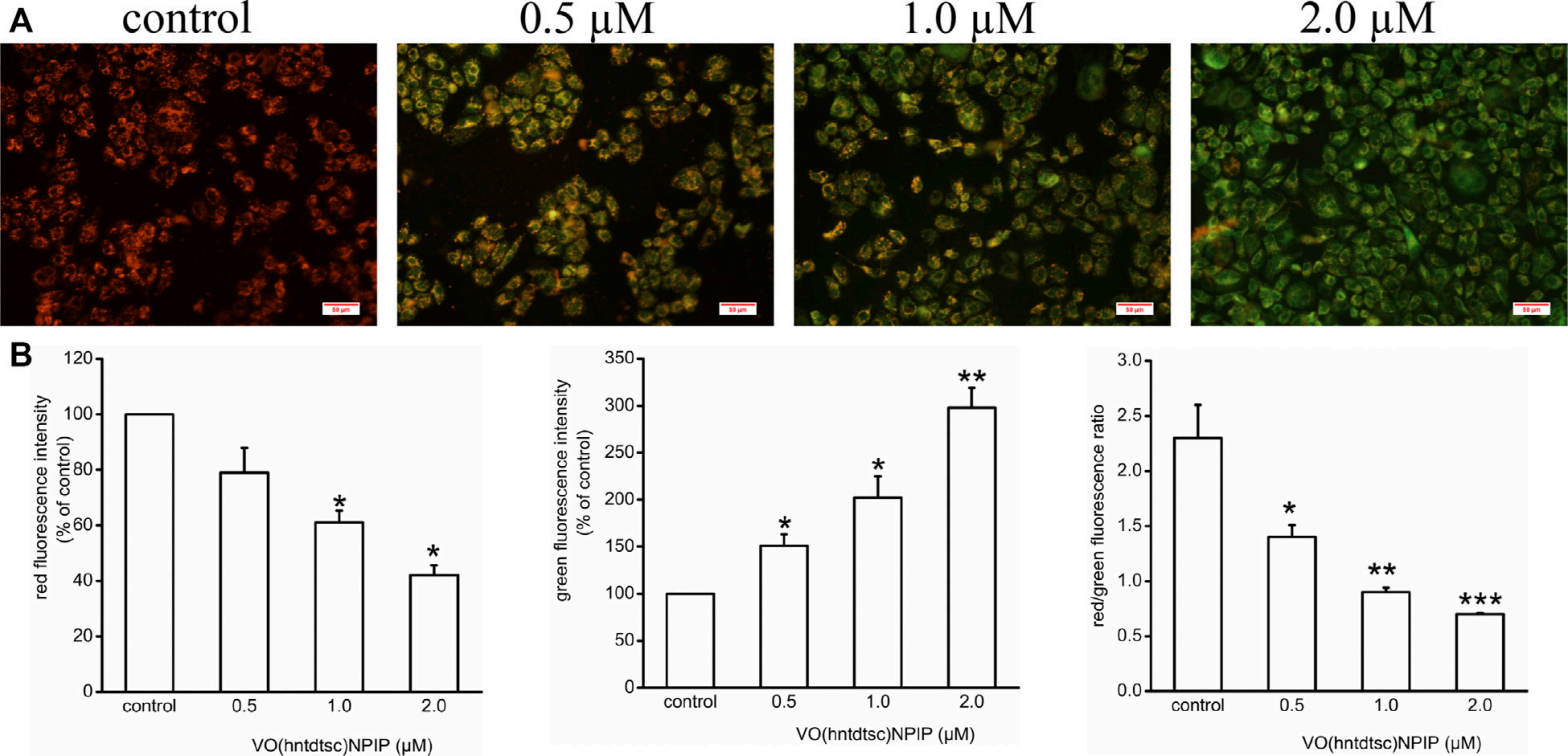
Effects of VO(hntdtsc)NPIP on mitochondrial membrane depolarization (MMP) in HeLa cells. **(A)** The changes of MMP of HeLa cells were detected by JC-1 probe. HeLa cells were treated with various concentrations (0, 0.5, 1.0, and 2.0 µM) of VO(hntdtsc)NPIP for 48 h (× 400). **(B)** Histograms displayed the red/green fluorescence intensity and red/green fluorescence ratio. Data are represented as mean ± SD of three independent experiments. *p < 0.05, **p < 0.01 or ***p < 0.001 compared to the control group.

To lucubrate the effects of VO(hntdtsc)NPIP on apoptosis, the changes of MMP in HeLa cells were detected by JC-1 dye, which was a fluorescent probe that has been widely used in the detection of MMP (Elefantova et al., 2018). As shown in Figure 3, compared with the control group, the red fluorescence intensity decreased (*F =* 36.08, *p* < 0.05) and the green fluorescence intensity increased (*F =* 55.22, *p* < 0.01) in a dose-dependent manner after VO(hntdtsc)NPIP treatment on HeLa cells, and the red/green fluorescence intensity ratio significantly decreased (*F=* 15.14, *p* < 0.01) as well, indicating that VO(hntdtsc)NPIP could effectively induce the decrease of MMP in HeLa cells.

#### Evaluation of the Expression of Apoptosis-Related Proteins

**FIGURE 4:**
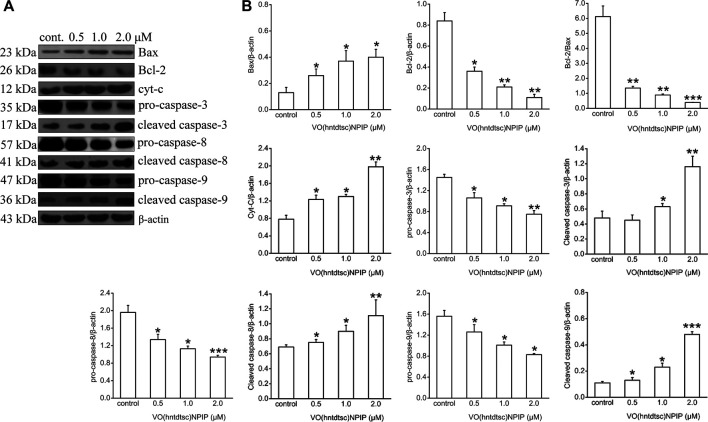
Mitochondria-dependent-apoptosis-related proteins were detected using western blotting; HeLa cells were treated with VO(hntdtsc)NPIP at different concentrations (0.5, 1.0, and 2.0 μM) for 24 h. The quantification of the proteins was exhibited following the immunoblotting analysis. **(A)** The expressions of Bax, Bcl-2, cytochrome c, pro-caspase-3, cleaved-caspase-3, pro-caspase-8, cleaved-caspase-8, pro-caspase-9, and cleaved-caspase-9 were determined by western blotting using specific antibodies. **(B)** The density ratio of Bax, Bcl-2, cytochrome c, pro-caspase-3, cleaved-caspase-3, pro-caspase-8, cleaved-caspase-8, pro-caspase-9, and cleaved-caspase-9 to β-actin, after treatment with VO(hntdtsc)NPIP. Data are represented as mean ± SD of three independent experiments. *p < 0.05, **p < 0.01 or ***p < 0.001 compared to the control group.

Apoptosis is an important continuous process of destruction of undesirable cells during the development or homeostasis in multicellular organisms. It has also been known that cancer was caused by the disruption of cellular homeostasis between cell death and cell proliferation, and compounds that can induce apoptosis are considered to have potential as anticancer drugs (Verquin et al., 2004). To further illustrate the mechanism of VO(hntdtsc)NPIP on inducing apoptosis, the expression of apoptosis-related proteins was detected by western blot assay. As shown in Figure 4, VO(hntdtsc)NPIP significantly increased the expression of Bax, cytochrome c, cleaved caspase-3, cleaved caspase-8, and cleaved caspase-9 (*F =* 16.22, *p* < 0.05; *F =* 54.32, *p* < 0.001; *F =* 25.54, *p* < 0.01; *F* = 34.68, *p* < 0.001; *F =* 40.63, *p* < 0.001) and decreased the expression of pro-caspase-3, pro-caspase-8, and pro-caspase-9 (*F =* 15.68, *p* < 0.05; *F =* 16.39, *p* < 0.01; *F =* 11.36, *p* < 0.05) with concentrations increasing (0, 0.5, 1, and 2 µM) accordingly. Simultaneously, VO(hntdtsc)NPIP obviously decreased the expression of Bcl-2 (*F =* 33.12, *p* < 0.001) and reduced the ratio of Bcl-2/Bax (*F =* 86.74, *p* < 0.001) with concentrations increasing (0, 0.5, 1, and 2 µM), which further proved that VO(hntdtsc)NPIP inducing the apoptosis of HeLa cells was related to mitochondrial apoptosis. Combined with the mitochondrial depolarization results described above, these results suggested that VO(hntdtsc)NPIP induced apoptosis via mitochondrial apoptosis pathway.

### Antitumor Effect of VO(hntdtsc)NPIP *In Vivo*

#### Acute Toxicity Study on Kunming Mice

**FIGURE 5:**
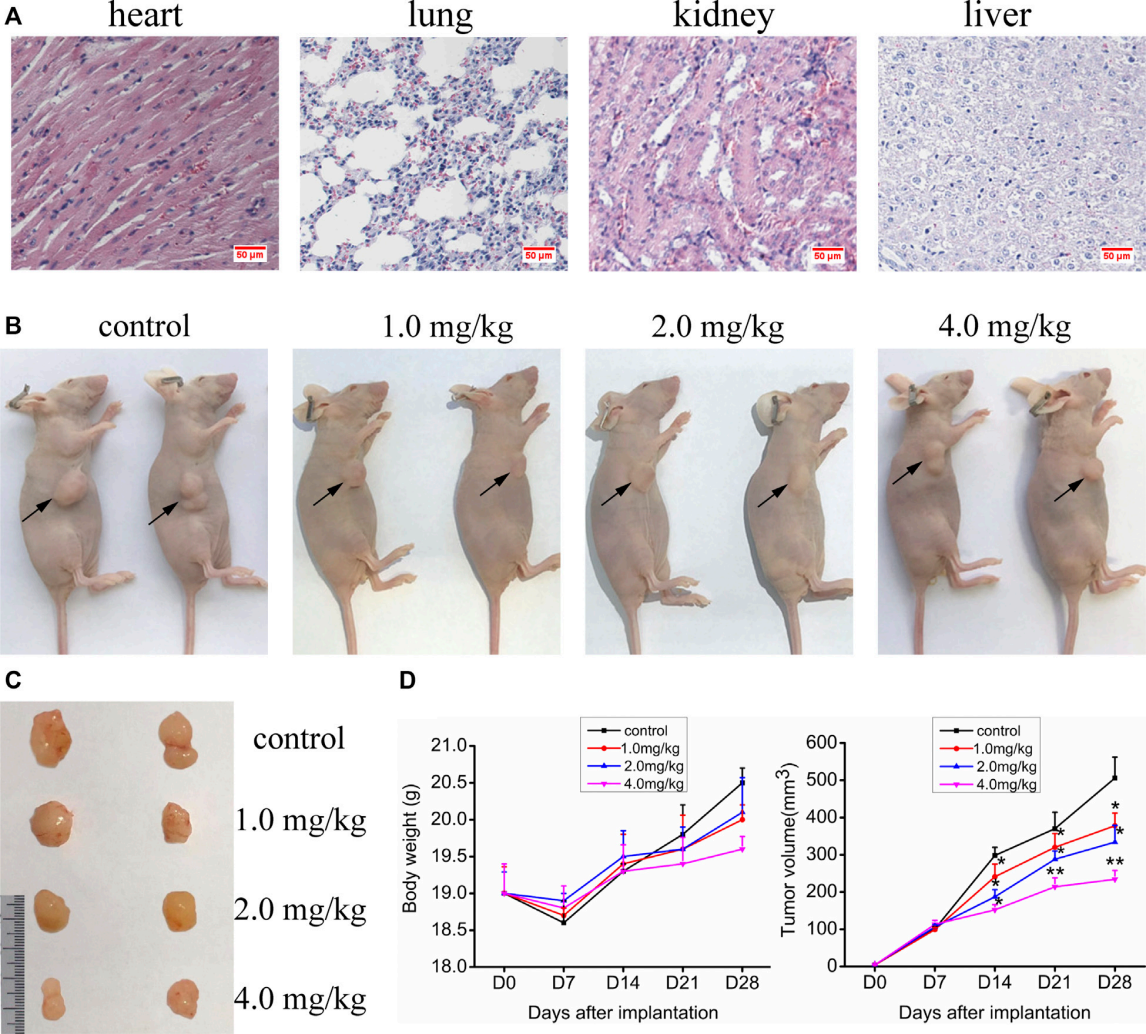
**(A)** H&E staining was performed on heart, liver, kidney, and lung tissue sections of mice after a dose of 4.0 mg/kg of VO(hntdtsc)NPIP treatment (× 400). **(B)** Nude mice bearing HeLa cells at the 28th day after transplantation. **(C)** Nude mice xenograft specimens at the 28th day after inoculation of HeLa cells. **(D)** Growth curves of the average body weight and tumor volume within each experimental group on the 0, 7th, 14th, 21^st^, and 28th days after tumor transplantation. Tumor volume was calculated using the following formula: width2 × length × 0.5. *p < 0.05 or **p < 0.01 compared to the control group.

The experiments *in vitro* mentioned above indicate that VO(hntdtsc)NPIP has significant antiproliferative and apoptosis-induced efficacy on HeLa cells. We then verified its activity in *in vivo* animal model. In order to confirm the nontoxic and effective dosage, we firstly tested the acute toxicity of VO(hntdtsc)NPIP on sixty Kunming mice according to OECD guidelines. The results showed that in the control group, all the indicators were basically normal in 14 days, including normal diet, good mental state and active, elastic skin, smooth back hair closed to the epidermis, and normal weight gain. With the dosage of VO(hntdtsc)NPIP increasing, the toxic reaction symptoms of the mice became more and more obvious, revealing a good dose-toxicity dependence relationship. Detailedly, when the mice were treated with VO(hntdtsc)NPIP after 24 h, one female mouse died at 8.0 mg/kg, four mice died at 16.0 mg/kg, and six mice died at 32.0 mg/kg, respectively. At the maximum dose of 64.0 mg/kg, all mice died within 4 h, and there was no significant difference between male and female mortality. The weight growth rates of the control group and the groups treated with VO(hntdtsc)NPIP at different dosages (4.0, 8.0, and 16.0 mg/kg) were 20.12%, 19.48%, 14.22%, and 3.13%, respectively, and the weight loss was 0.16% in the group treated with 32.0 mg/kg. In addition, according to the symptoms of the mice in the experiment, such as decreased autonomic activity, convulsion, imbalance of body, and diarrhea, it could be preliminarily estimated that VO(hntdtsc)NPIP might be relatively toxic to the central nervous system, neuromuscular system, and digestive system. It is worth noting that the LD_50_ value of VO(hntdtsc)NPIP was 24.26 mg/kg with 95% confidence interval [17.37, 34.91], which was nearly twice to that of the classic metal drug cisplatin, indicating that VO(hntdtsc)NPIP was less toxic and had the significance for further study. In addition, in the VO(hntdtsc)NPIP treated group at a dosage of 4.0 mg/kg, the Hematoxylin-Eosin (H&E) staining pathological sections collected from heart, lung, kidney, and liver showed no observable major organ-related toxicities (Figure 5A). Based on these experimental results, the relatively safe dose of 1.0, 2.0, and 4.0 mg/kg was selected for xenograft nude mice models *in vivo*.

#### Effects of VO(hntdtsc)NPIP on the Growth of HeLa Xenografts

**FIGURE 6:**
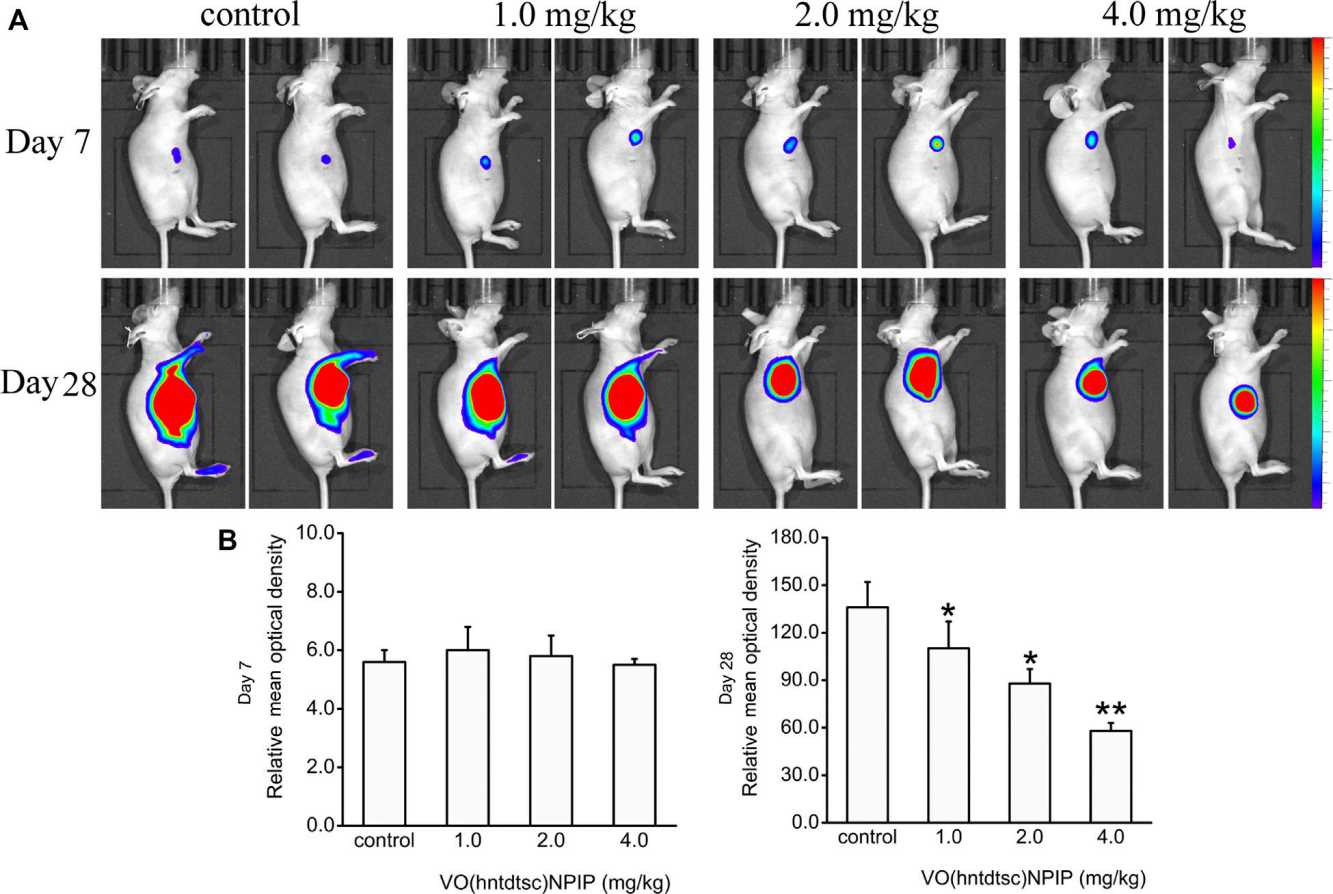
Fluorescence kinetics in HeLa xenografts in nude mice after 10-Gy irradiation. **(A)** Dynamical observation of tumor growth with *in vivo* imaging system on the 7th and 28th days, respectively, after different doses of VO(hntdtsc)NPIP treatment. **(B)** Effects of VO(hntdtsc)NPIP on mean optical density of HeLa xenografts in nude mice on the 7th and 28th days.*p < 0.05 or **p < 0.01 compared to the control group.

Up to date, overwhelming evidence demonstrates that oxovanadium complexes have great potential of antiproliferation against various tumor cells; however, studies *in vivo* are scarce, which perhaps is the key obstacle in the translational development of these compounds to drugs [15–19]. To further verify the inhibitory efficacy of VO(hntdtsc)NPIP against tumor growth *in vivo*, the HeLa cells xenograft models in nude mice were established successfully using tumor tissue block method, which could ensure the uniformity of tumor formation (Lee et al., 2018). On the 7th day after tumor transplantation, the volume of lumps all reached 100.0 mm^3^ and met the requirements of drug administration. Then, the nude mice were treated with VO(hntdtsc)NPIP by intraperitoneal injection in the doses of 1.0, 2.0, and 4.0 mg/kg every 3 days for a total of 7 times. And the body weight and tumor volume of nude mice on the 0, 7th, 14th, 21^st^, and 28th days after tumor transplantation were selected to statistical analysis. The tumor-bearing states and tumor anatomical specimens of nude mice in each group on the 28th day after tumor transplantation were shown in Figure 5B,C. The body weight growth curve of the nude mice showed that, compared with that before the transplantation, the average body weight of the nude mice in each group gradually increased on the 14th, 21^st^, and 28th days after transplantation (Figure 5D). In addition, tumor volume growth curve showed that VO(hntdtsc)NPIP could suppress the tumor growth rate in a dose-dependent manner and had a significant antitumor effect (Figure 5D). Moreover, in order to confirm the inhibitory effect of VO(hntdtsc)NPIP against HeLa cell xenografts growth and metastasis, dynamical observation of tumor growth *in vivo* imaging system was tested (Figure 6) and the results showed that, compared with the control group, VO(hntdtsc)NPIP decreased the relative light intensity of live imaging in nude mice in a dose-dependent manner, indicating VO(hntdtsc)NPIP could significantly delay the onset of tumor growth and inhibit visible tumor progression, which was consistent with the results shown in Figure 5D. In a word, the above results are consistent with the experimental results of antihuman cervical cancer HeLa cells proliferation *in vitro*, which fully proved that the complex VO(hntdtsc)NPIP has significant antiproliferative activity against HeLa cells.

#### H&E Staining of Tumor Tissues in Nude Mice Bearing HeLa Cells

**FIGURE 7:**
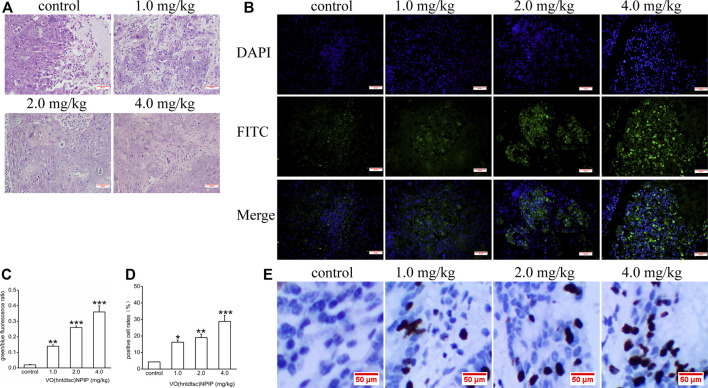
**(A)** H&E staining was performed on tumor tissues in nude mice bearing HeLa cells after various doses of VO(hntdtsc)NPIP treatment and a representative images have been displayed (× 200). **(B)** Apoptotic effect of VO(hntdtsc)NPIP on HeLa cells in xenograft nude mice was determined by TUNEL and DAPI assay. TUNEL (FITC, green) was used to mark fragmented DNA. DAPI (blue) was used to indicate the cell nuclei. The changes of fluorescence intensity were determined using TUNEL (FITC) + DAPI double staining after VO(hntdtsc)NPIP treatment (× 400). **(C)** Histograms display the green/blue fluorescence ratio. **(D)** Histograms display the positive cleaved caspase-3 cell rates of xenografts after various doses of VO(hntdtsc)NPIP treatment. **(E)** The expression changes of cleaved caspase-3 after VO(hntdtsc)NPIP treatment were detected using immunohistochemical staining. (× 400). Data are represented as mean ± SD of three independent experiments. **p < 0.01 or ***p < 0.001 compared to the control group.

Associations between the density of tumor cells in H&E-stained sections were shown in Figure 7A, suggesting that, in the control group, the tumor cells in the tumor tissue are numerous, densely arranged, basophilic, and hyperchromatic, and the atypia nuclei increased, presenting typical cancerous changes. While in the group with VO(hntdtsc)NPIP treatment in different doses (1.0, 2.0, and 4.0 mg/kg), opposite results were observed: the tumor cells were significantly decreased, the cell spacing was widened and the arrangement was looser, and karyopyknosis and the varying degrees of degenerative necrosis had occurred, indicating that VO(hntdtsc)NPIP can effectively induce the apoptosis of HeLa cells in xenograft models in nude mice.

#### Effects of VO(hntdtsc)NPIP on the Apoptosis in HeLa Xenografts

As for cells apoptosis, DNA endonuclease can be active with cutting the genomic DNA in the nucleosomes and exposing 3'-OH terminal (Madeo et al., 1997). And the fragmentation of nuclear DNA at the late stage of apoptosis can be detected by FITC labeled TUNEL cell apoptosis detection kit (Wadskog et al., 2004). DAPI, a blue fluorescent dye that binds strongly to DNA, can pass through the intact cell membrane and bind to the AT base pair position of the double-stranded DNA groove, staining apoptotic and nonapoptotic cells to blue so that the number of DNA can be detected (Madeo et al., 1999). To further examine VO(hntdtsc)NPIP-induced apoptosis of HeLa cells in xenograft nude mice, we investigated the colocalization of green fluorescence and blue fluorescence by TUNEL (FITC)/DAPI staining, and the proportion of apoptotic cells after VO(hntdtsc)NPIP treatment was detected (Park and Lee, 2010). As shown in Figure 7B,C, after VO(hntdtsc)NPIP treatment, the green fluorescence staining with TUNEL (FITC) was enhanced, and the green fluorescence/blue fluorescence ratio increased in a dose-dependent manner (*F =* 133.14, *p* < 0.001), indicating VO(hntdtsc)NPIP increased the apoptosis rate of HeLa cells. To further verify the efficacy of VO(hntdtsc)NPIP on the apoptosis in HeLa xenografts, the expression level of cleave caspase-3 was detected by immunohistochemical staining. As shown in Figure 7D,E, VO(hntdtsc)NPIP remarkably increased the expression of cleave caspase-3 in a dose-dependent manner (*F =* 61.77, *p* < 0.01), suggesting that VO(hntdtsc)NPIP could effectively induce the apoptosis of HeLa cells in xenograft nude mice.

#### Effects of VO(hntdtsc)NPIP on the Expression of Ki-67 and p16 in HeLa Xenografts

**FIGURE 8:**
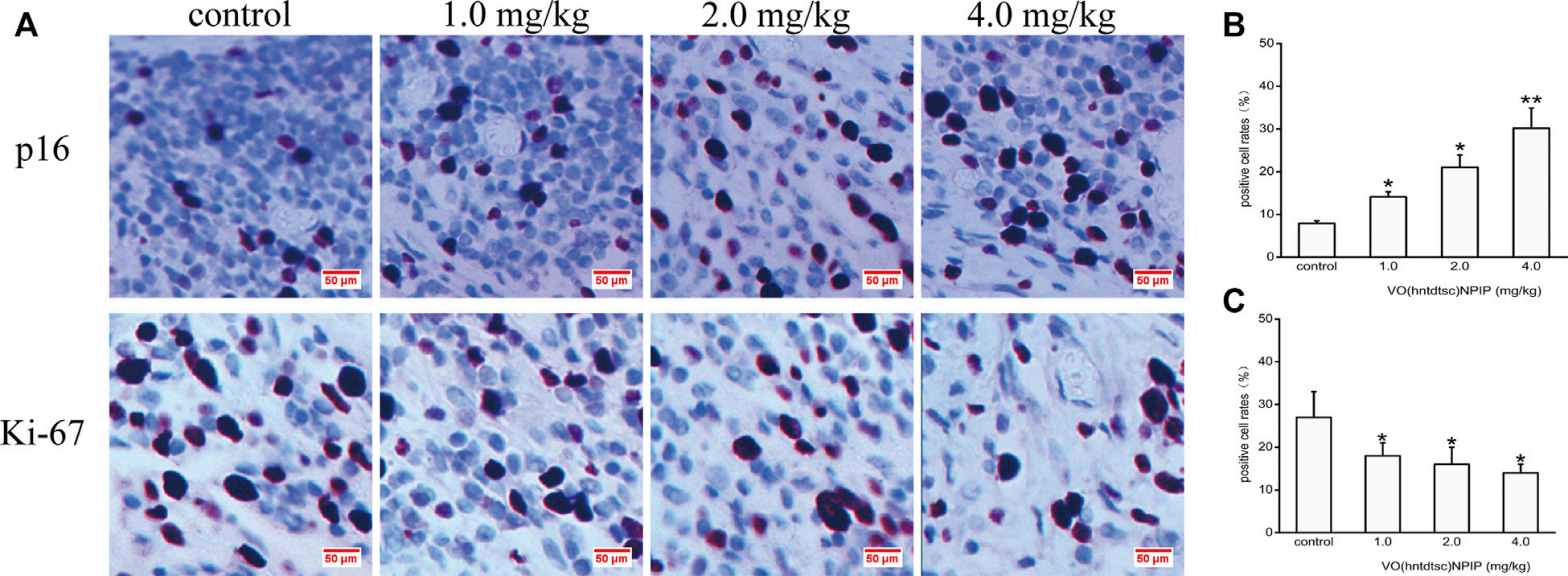
Expression of p16 and Ki-67 in HeLa xenografts in nude mice was determined by immunohistochemical staining. **(A)** The expression changes of p16 and Ki-67 after VO(hntdtsc)NPIP treatment were detected using immunohistochemical staining. (× 400) **(B)** Histograms display the positive p16 cell rates of xenografts after various doses of VO(hntdtsc)NPIP treatment. **(C)** Histograms display the positive Ki-67 cell rates of xenografts after various doses of VO(hntdtsc)NPIP treatment. All data are represented as mean ± SD of three independent experiments. *p < 0.05 or **p < 0.01 compared to the control group.

To further explore the effects of VO(hntdtsc)NPIP on cell proliferation in HeLa xenografts, immunohistochemical staining was conducted and the results was shown in Figure 8, revealing that the VO(hntdtsc)NPIP treatment significantly reduced the cell positive rate with Ki-67 staining (*F =* 140.22, *p* < 0.001) and enhanced the cell positive rate with p16 staining (*F =* 79.55, *p* < 0.01), suggesting that VO(hntdtsc)NPIP obviously inhibited the proliferation index of HeLa cell xenografts and reduced the malignant degree of the transplanted tumor.

## Discussion

In this study, a comprehensive antitumor activity study of VO(hntdtsc)(NPIP) on cervical cancer HeLa cells *in vitro* and *in vivo* was conducted. Cytotoxicity assays on HeLa, BIU-87, SPC-A-1, SGC-7901, HT-29, PANC-1, and HepG2 cell lines showed that VO(hntdtsc)(NPIP) had an obvious antiproliferative activity against the above-mentioned seven common human cancer cell lines, particularly HeLa cells in a good concentration-dependent and time-dependent manner. Cell cycle is a vital process by which a cell leads to duplication, and disorders of the cell cycle regulation may lead to tumor formation. Growing evidence has demonstrated that many antitumor drugs exert their antitumor efficacy by regulating the cell cycle (Niknejad et al., 2016; Ling et al., 2018). The successful progression of the cell cycle mainly depends on three regulatory factors, including cyclins, cyclin-dependent kinases (CDKs), and cyclin-dependent kinase inhibitors (CKIs) (Satyanarayana and Kaldis, 2019). Studies have shown that cyclin-CDKs complexes, such as cyclin D1-CDK4/6 complexes, mediate the transformation process from G1 phase to S phase, and p16 proteins, as one of the main members of CKIs, are also an important regulatory factor for the transformation from G1 phase to S phase (Hong et al., 2018). The overexpressed p16 could compete with cyclin D1 for CDK4, thereby inhibiting the formation and activity of cyclin D1-CDK4 complex, leading to the dephosphorylation and inactivation of p-Rb and causing G1 phase arrest of the cell (Sherr and Roberts, 1999). In terms of mechanism, the cell cycle-phase distribution was analyzed by flow cytometry with PI staining and the expression of associated cell cycle regulatory proteins was detected using western blot assay, and we found that VO(hntdtsc)NPIP could obviously arrest HeLa cells cycle at G0/G1 phase via p16-cyclin D1-CDK4-p-Rb pathway. In view of the obvious antiproliferation effect of VO(hntdtsc)NPIP *in vitro*, we further explored the antiproliferation effect of VO(hntdtsc)NPIP *in vivo*. Ki-67, a nuclear antigen associated with cell mitosis, mainly labeled cells in a proliferation cycle other than G0 phase, and the higher the positive rate of the marker is, the faster the tumor growth is and the higher the degree of malignancy is (Qiao et al., 2016). P16, a tumor suppressor, can prevent the cells from entering the S phase by inhibiting the activity of CDK4 and has become an important target for the development of antitumor drugs on cell cycle inhibition (Kobierzycki et al., 2018). Further exploration on the cell proliferation effect of VO(hntdtsc)NPIP in HeLa xenografts revealed that VO(hntdtsc)NPIP obviously inhibited the proliferation index of HeLa cell xenografts and reduced the malignant degree of the transplanted tumor, which further verified the results of experiments *in vitro* and suggested that VO(hntdtsc)NPIP might regulate the p16-cyclin D1-CDK4-p-Rb pathway while reducing the proportion of cells entering the S phase, suppressing the synthesis of DNA and chromatin structural proteins, interfering with the mitotic process of HeLa cells, and ultimately leading to apoptosis or necrosis of HeLa cells.

Apoptosis is a programmed progress, and the unlimited proliferation of tumor cells caused by the dysregulation of apoptosis is one of the important causes of tumorigenesis. It is generally believed that mammalian apoptosis occurs mainly through two pathways, including the death-receptor-induced extrinsic pathway and the mitochondria-apoptosis-mediated intrinsic pathway (Kiraz et al., 2016). In the mitochondria-apoptosis-mediated intrinsic pathway, mitochondria not only serve as the regulatory center of cell apoptosis, but also is a key link in the cascade of apoptosis (Nagy et al., 2015). The assessment of mitochondrial membrane potential (MMP) in intact cells could yield information which is necessary for the evaluation of their physiopathological conditions (Seppet et al., 2009). The loss of MMP often takes place during the induction of neoplastic cell apoptosis (Ortiz-Lazareno et al., 2014). In this study, we have preliminarily found that VO(hntdtsc)NPIP could effectively induce apoptosis of HeLa cells. To lucubrate the effects of VO(hntdtsc)NPIP on apoptosis, the changes of MMP in HeLa cells were detected and the result showed that VO(hntdtsc)NPIP could effectively induce the decrease of MMP in HeLa cells. In addition, the oxidative-damage-induced mitochondrial-dependent apoptosis pathway has attracted more attention in the study of the mechanism of metal antitumor drugs (León et al., 2014). Furthermore, growing evidence suggests that the induction of oxovanadium complexes on the production of oxygen free radicals such as reactive oxygen species (ROS) can cause oxidative stress damage in tumor cells (Maurya et al., 2010). Consistently, we also found that VO(hntdtsc)NPIP could significantly induce the increase of ROS levels in HeLa cells in a dose-dependent manner. To further illustrate the mechanism of VO(hntdtsc)NPIP on inducing apoptosis, detetion of apoptosis-related proteins expression was essential. The Bcl-2 family of proteins plays a key regulatory role in the opening of the mitochondrial intimal permeability transition pore (PT), including proapoptotic proteins such as Bax and antiapoptotic proteins such as Bcl-2 (Martinou and Youle, 2011). The opening of PT pore causes the change of MMP, which eventually leads to the destruction of MMP and the release of cytochrome c into the cytoplasm, which interacts with the apoptotic protease activator (Apaf-1) and the precursor of caspase-9 to form a complex, activating the downstream caspase (e.g., caspase 3) and eventually resulting in the occurrence of apoptosis cascade reaction (Gross, 2016). Overwhelming evidence has proved that the general mechanisms of vanadium complexes for antitumor action involve opening of mitochondrial permeability transition pores (PT) resulting in cancer cell apoptosis (Zhao et al., 2010). In this study, we found that VO(hntdtsc)NPIP significantly increased the expression of Bax, cytochrome c, cleaved caspase-3, cleaved caspase-8, and cleaved caspase-9 and decreased the expression of pro-caspase-3, pro-caspase-8, and pro-caspase-9 in a dose-dependent manner accordingly. Similarly, VO(hntdtsc)NPIP obviously decreased the expression of Bcl-2 and reduced the ratio of Bcl-2/Bax with concentrations increasing, which further demonstrated that VO(hntdtsc)NPIP induced apoptosis via mitochondrial apoptosis pathway. Consistently, VO(hntdtsc)NPIP could effectively increase the expression of cleaved caspase-3 and induce the apoptosis of HeLa cells in xenograft nude mice.

To fully understand the mechanism involved in the regulation of cell cycle and induction of apoptosis by VO(hntdtsc)NPIP, further investigation needs to be carried out. All the above initial observations are indeed encouraging to contemplate in-depth studies in future.

## Conclusion

In conclusion, the anticancer activity and the mechanism of the novel oxovanadium complex VO(hntdtsc)NPIP on cervical cancer HeLa cells were further evaluated *via* several biological assays *in vitro* and *in vivo*. The preliminary studies of anticancer activity showed that VO(hntdtsc)NPIP significantly inhibited the proliferation of human cervical cancer HeLa cells, resulting in arresting cell cycle at G0/G1 phase through the p16-cyclin D1-CDK4-p-Rb pathway, which was demonstrated by upregulating the expression of p16 and downregulating the expression of cyclin D1, CDK4, and p-Rb and inducing cell apoptosis through the mitochondrial-dependent apoptosis pathway, which was further illustrated by downregulating the expression of Bcl-2/Bax ratio, pro-caspase-3, pro-caspase-8, and pro-caspase-9, and upregulating the expression of cytochrome c, cleaved caspase-3, cleaved caspase-8, and cleaved caspase-9, the loss of MMP, and the change of cellular morphology. Furthermore, the antitumor efficacy was verified in cervical cancer xenograft mice models *in vivo*. In accordance with the *in vitro* results, *in vivo* experiments revealed that VO(hntdtsc)NPIP significantly inhibited the cancer cell growth and induced the cancer apoptosis. As a bold conjecture, considering the IC_50_ value of VO(hntdtsc)NPIP against HeLa cell lines was only one-fifth such that, of cisplatin (5.54 ± 0.81 μM), an interesting project to make a systematic comparison between VO(hntdtsc)NPIP and cisplatin in HeLa cells could be conducted in the future. And the antiproliferation efficacy of VO(hntdtsc)NPIP in HeLa cells would be further verified and accelerate the transformation pace of synthetic compounds to clinical drugs. Collectively, our research dmonstrated that VO(hntdtsc)NPIP possessed a great potential in the treatment of cervical cancer and was worthy of continuous development. We believe that this study could provide an important impetus for the research and development of the anticancer potential of oxovanadium complexes; especially, VO(hntdtsc)NPIP is expected to be developed as a lead compound and further transformed into a new anticancer drug. Further investigation into the development of efficient anticancer agents based on these oxovanadium complexes is underway in our group.

## Data Availability

The original contributions presented in the study are included in the article/Supplementary Material; further inquiries can be directed to the corresponding authors.
